# Possibility of Using Wind Turbine Waste in Particleboard Manufacturing

**DOI:** 10.3390/polym16091210

**Published:** 2024-04-26

**Authors:** Adam Derkowski, Dorota Dziurka, Ryszard Antonowicz, Monika Chuda-Kowalska, Radoslaw Mirski

**Affiliations:** 1 Department of Mechanical Wood Technology, Faculty of Wood Technology, Poznań University of Life Sciences, Wojska Polskiego 38/42, 60-627 Poznań, Poland; adam.derkowski@up.poznan.pl (A.D.); dorota.dziurka@up.poznan.pl (D.D.); 2 Faculty of Civil Engineering, Wrocław University of Science and Technology, Wybrzeże Wyspiańskiego 27, 50-370 Wrocław, Poland; ryszard.antonowicz@pwr.edu.pl; 3 Institute of Structural Analysis, Faculty of Civil and Transport Engineering, Poznan University of Technology, pl. Sklodowskiej-Curie 5, 60-965 Poznań, Poland; monika.chuda-kowalska@put.poznan.pl

**Keywords:** blades, polymer waste, chip board mechanical properties

## Abstract

Recent reports indicate that the development of electricity generation using wind turbines will continue to grow. Despite the long service life of wind turbine blades, their technological life comes to an end at a certain point. Currently, there is no industrial method for recycling them, and the proposed solutions need to consider a complete and comprehensive approach to this material. In many countries, these blades are stored in special landfills and await proposals for rational recycling. It has been proposed that this recyclable yet still troublesome raw material be used in building sheathing boards. Sheathing boards used in the construction industry have a relatively long lifecycle. Three types of polymer chips and two resins, i.e., PF and MUF, were used in the study. The boards’ quality was assessed per the standards specified for particle boards. The resulting boards were characterized by strengths above 20 N/mm^2^ and an elastic modulus close to 4000 N/mm^2^. Slightly better results were obtained with the MUF resin.

## 1. Introduction

The overarching goal of policy initiatives, the so-called Green Deal [1], guiding the EU (European Union) countries on the path of environmental transition is to achieve climate neutrality by 2050. The areas covered are industry, agriculture, transport, and energy, as well as their impact on the environment and climate change. Regarding energy generation and use, the initiatives seek to disseminate and develop cleaner energy sources and decarbonize existing ones.

Wind energy is a part of the energy sector that fits perfectly with the above initiatives and activities. Virtually free, widely available and inexhaustible, wind energy is the second most intensively used source for electricity production after the gravitational energy of water. The rapid growth of wind farm electricity generation began at the beginning of the 21st century. Today, the United States and China lead the way in wind turbine electricity generation, while Denmark, Germany, Spain and Ireland lead the way in Europe. In the case of Denmark, almost 50% of the country’s electricity demand comes from wind farms, mainly located at sea. A turbine’s blade design must withstand various loads of different natures, mainly cyclic and fatigue. Hence, composite structures are the most popular.

Currently, there is a trend towards constructing more prominent and larger turbine diameters [2], which increases power output and reduces the unit cost of energy production. However, this trend is accompanied by increased rotor weight and the need to increase blade stiffness. A sufficiently high stiffness-to-weight blade ratio is one of the most critical parameters. Hence, the most commonly used reinforcement in composite blade sheathing and internal blade supports are various glass or carbon fibres bonded with synthetic resin. There are many variations in rotor blade manufacturing technology. However, mould casting methods are still the most popular, varying in the type of fibres and resins used and the processing method. In addition to carbon and glass fibres, aramid fibres, basalt fibres and combinations of these are used, and attempts are also being made to use vegetable fibres. Thermosetting polyester resins usually perform the matrix function of the fibres. Other polymers and thermoplastics are also used as options.

The primary load on a propeller is wind and gravity loading, and the average service life is 20–25 years. Despite the high damage resistance of the composites, there is progressive degradation of the blades’ components after this period, usually due to fatigue loading—the number of load cycles usually exceeds 100 million. A fundamental problem with wind turbines is their durability and how to deal with the waste at the end of their life. Composite propeller blades are not easy to recycle because it is difficult to separate the individual components. Like other composites, the processes used to recycle wind turbines can be mechanical, thermal (e.g., pyrolysis) or chemical [3,4,5]. Mechanical shredding is relatively cheap but is accompanied by the release of harmful dust. Chemical processes are the most energy-intensive [6]. Alternative waste management methods include landfilling, co-incineration in cement plants or manufacturing small architectural objects [7,8].

Although the literature on how to recycle composite blades is abundant, with more technologies being investigated and implemented [9,10,11], the problem of disposing of waste blades will grow [12,13,14], with increasing demand for electricity [15]. Globally, the waste mass is estimated to grow to reach about 3.3 million tons per year in 2050. Some countries producing electricity with wind turbines will soon face difficult decisions regarding upgrading or possibly closing wind farms. Based on a detailed literature review, the work of [16,17] presents a multi-criteria methodology, strengthening the decision-making process for so-called repowering.

The problem of the recycling of used composite materials affects the RES (Renewable Energy Sources) industry—PV (photovoltaic) cells are also difficult to recycle [18]—and many other industries, such as the automotive and aerospace industries [19]. This reasoning is leading researchers and manufacturers to look for new and improved methods of recycling composites and recovering fibres, including wind turbine blades. There is quite a lot of work dedicated to this area [20,21,22,23,24,25,26,27,28,29,30]. Numerous studies and tests are also being conducted to produce new generations of composites. The emphasis is on creating innovative materials, preferably containing renewable, easily recyclable, biodegradable components of natural origin that are environmentally friendly. This applies to almost all areas of industry, including wind power. Leading windmill blade manufacturers are implementing new and, as they assure us, fully recyclable composites, including those based on thermoplastic polymers [31,32].

Industries that commonly produce other types of composite structures include the furniture and construction industries and, more specifically, manufacturers of all kinds of panel materials. The most significant volume of their products comprise single or multi-layer boards, panels, and laminates. The board industry is developing dynamically; new solutions are being searched for, and those already developed are being improved. In addition to traditional particleboard, manufacturers and researchers are increasingly interested in new innovative composites in which matrices, fillers and additives are changed. For example, there is a trend towards changing the composition of fillers from high-class to readily available, low-cost and biodegradable fillers. Thus, many plant-based fibres are used as a substitute for wood chips in the particleboard industry for construction and furniture. These fibres have a reinforcing or filling function or an additive role. They are usually extracted from many plant species’ seeds, stems, leaves, grasses or straws. The resulting biocomposites are environmentally friendly, and their use is also supported by the availability of a considerable amount of raw material (according to geographical location). In a review paper by Lee et al. [33], a characterization of particleboard markets and current trends in various countries worldwide is included. Many studies have confirmed the beneficial effects of additives and organic fillers on the physical and strength properties of the final products. Further research is needed on the interaction between the polymer medium and the fibres, which can be controlled by appropriate treatment of the composite components. The work by Mirski et al. and Banaszak et al. [34,35,36] addressed this topic. The usefulness of straw fibres from rye, triticale and rape and other lignocellulosic materials as a filler in three-layer composite panels has been investigated. Optimum panel configurations have been identified for the matrix and straw filler used. Mirski et al. [37] studied the effect of moisture content on OSB (Oriented Strand Board) filled with fine chips in the core layer. They determined that their maximum content did not deteriorate strength, stiffness, or dimensional stability. Borysiuk et al. [38], on the other hand, determined the optimum ratio of wood chip filler content on the density and density profile of popular WPC panels produced by extrusion and compression moulding. Gozdecki et al. [39] investigated panels filled with wood chips from milling three-layer particleboard bonded with polypropylene. The mechanical properties of such boards do not differ significantly from the original particleboard, and the idea of using so-called circularized products is currently promoted and very topical. The works of Gozdecki and Wilczyński [40,41] contain the results of tests on boards with a filler in the form of different types of wood floor. An older paper by Gozdecki et al. and Kociszewski [42,43] investigated the relationship between the size and shape of industrial chips and the strength parameters of three-layer wood-polymer boards. Both chip size and mass proportion significantly affected the board’s strength and stiffness in bending and tensile. Similar studies have also been conducted in other sectors of the plastics industry, such as in manufacturing new generations of rubbers [44,45]. The suitability of using fillers made from cereal straw and other plants was investigated; their beneficial effect on the mechanical and physical properties of the new plastic was found.

This study is concerned with using wind turbine blade waste to produce sheathing boards. The ground blades were mixed with wood chips at a ratio of 40/60. The promising results of previous research [46] have led the authors of this paper to produce and investigate further, more durable and weather-resistant composite panels. The boards, made of a wood particle–polymer–glue mixture, were tested primarily for their mechanical properties and their resistance to water. 

## 2. Materials and Methods

The decision was made to use in the research industrial pine chips in the inner layer of the furniture particleboard. Such chips have been successfully used in producing so-called construction boards, intended to compete with OSB, by the Pfleiderer company in Wieruszów (Pfleiderer Wieruszów Sp. z o.o., Wieruszów, Poland). These boards have adopted the trade name MFP (Multi-Functional Panel). Chips of this type show linear dimensions falling within 5 mm/30 mm—length, 0.5 mm/3 mm—width, 0.2 mm/0.8 mm—thickness [34,37]. The chips for the middle layer were obtained from Swiss Krono (SWISS KRONO Sp. z o.o., Żary, Poland); however, most plants produce sizes (linear dimensions) of these chips falling within these ranges.

Approximately 40 tonnes of material were generated during the deconstruction of wind turbines, and it is assumed that 14,000 propellers will be replaced in Europe. The majority of these can be recycled, providing around 60,000 tonnes of shredded polymer composite. Whether it is the concrete structure or the blades themselves, the material is firstly defragmented into a form that can be transported effortlessly. The defragmentation process is relatively simple, as the propellers are cut up relatively slowly into smaller pieces using hand tools. The process is carried out on mats so that the resulting shavings do not enter the surrounding area during cutting. Propellers are often built on wood ribs and foam infill, over which carbon fibre is applied as the main body and glass fibre as the outer skin. The fibres are in the form of mats, which are bonded together with epoxy resin. Several layers are applied in this way. When cutting, i.e., after removing the wooden parts and polyurethane foams, a structure composed of glass/carbon fibres coated with epoxy resin forms a uniform ‘material’. Therefore, the grinding process is very energy-intensive, and it is almost impossible to separate these fractions. Grinding into fine fractions already occurs in specialised plants that protect their unique solutions. Thanks to the closed grinding process and the filter system, the process is safe in maintenance and for the environment. All caught fine particles are returned to the resulting dimensionally uniform granules.

**Figure 1:**
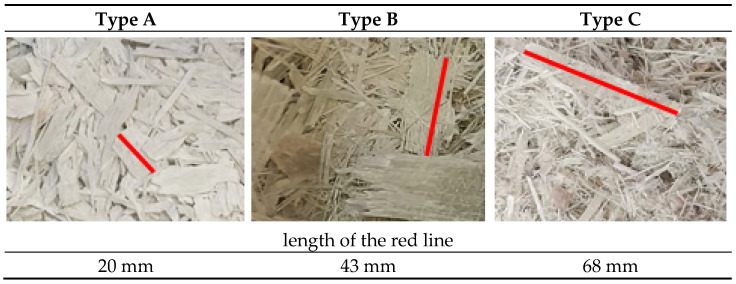
Appearance and dimensional relationships of the different types of propeller millings.

Shredded propeller blades with different linear dimensions were supplied by ANMET (Szprotawa, Poland). The other generic groups were described with letters A to C; their appearance is shown in Figure 1. The thickness of all fractions was similar and ranged from 0.1 mm to 1.2 mm (mean = 0.7 mm, SD = 0.28 mm). The resulting material of each fraction was sieved through a 0.5 × 0.5 mm mesh sieve to remove very fine and dusty facies. The material obtained from the shredded propellers was referred to as polymer chips instead of pine chips.

The good results of particle-polymer boards obtained in earlier studies indicate the possibility of producing furniture boards in which the middle layer is partially substituted with polymer chips [46], which may be a rationale for trying to make structural boards with higher mechanical properties. For this reason, it was decided to increase the density to 880 kg/m^3^ and to utilize resins used in the production of wood materials with increased resistance to weathering, i.e., phenol-formaldehyde resin (PF) and melamine-urea-formaldehyde resin (MUF).

Semi-technical conditions (only the mat t is formed by hand) were used to manufacture single-layer polymer particle boards with an assumed thickness of 15 mm. Polymer chips with a moisture content of 0.5% ± 0.1% were mixed with pine chips with a moisture content of 2.76%, in a ratio of 40% polymer chips and 60% pine chips. An adhesive was pneumatically applied to the material prepared in this way at a rate of 8% by dry weight of the adhesive to dry weight of the wood–polymer mixture. The resin used in the tests was MUF resin (the commercial symbol 410) and PF resin (the commercial symbol R1010). Both resins were supplied by Silekol (Silekol Sp. z o.o., Kędzierzyn-Koźle, Poland). A 20% ammonium nitrate solution was introduced into the MUF resin immediately before the adhesive was applied to the chips at 1.5% by the hardener’s dry weight to the adhesive resin’s dry weight. The dry weight of the PF resin was 49.5%, and that of the MUF was 75.4%. Both resins were used at commercial concentrations, and in the case of the MUF resin, there was only a slight decrease in concentration due to the use of the hardener. The boards were pressed at a specific pressure of 3.1 MPa for 450 s at a heating plate temperature of 185 °C. It was decided to refer to the boards produced in this way as wood-polymer (WP) boards, as opposed to WPC boards in which thermoplastic polymers form the matrix for the lignocellulosic material.

The manufactured board formats were air-conditioned for two weeks at 20 ± 2 °C and 60 ± 5% humidity. The moisture content of the boards after pressing was 2.9%/3.1%, while after the conditioning period, it was 5.8% ± 0.3%. Samples for testing were prepared from the boards in this way, in the amount of minutes. Twelve specimens were used for each test, except for the density profile test. The density profile was carried out on three samples.

After the conditioning, the produced boards were tested in terms of the following parameters according to the relevant standards:

- bending strength (MOR) and modulus of elasticity (MOE) according to EN 310 [47];
- internal bond (IB) according to EN 319 [48];
- thickness swelling (TS) after 24 h according to EN 317 [49] and water absorption (WA).

The mechanical properties were measured using a TinusOlsen 10K testing machine (Tinius Olsen Ltd., Salfords, UK), and the density profile using a Grecon Dax6000 (Fagus-GreCon Greten GmbH & Co. KG, Alfeld/Hanover, Germany).

The results obtained were analysed statistically and compared with those of previous studies. Statistica software version 13.0 (Version 13.0, StatSoft Inc., Tulsa, OK, USA) was used for statistical analysis.

## 3. Results and Discussion

**Figure 2:**
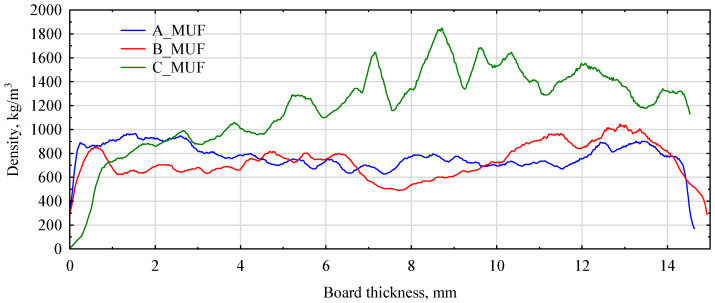
Density profiles of particleboards bonded with MUF resin and 40% polymer chips.

An attempt was made to assess the quality of the manufactured boards on the basis of their density profile. While this is a simple, quick and convenient method to evaluate the performance of the process line itself and the expected quality, primarily mechanical, of the manufactured particleboards of classical composition, the introduction of polymer chips into the wood chips greatly disturbs this view. From the presented profilographs made for boards manufactured with MUF resin, it can be seen that the introduction of fibres/shredded propeller parts results, especially when these fragments are relatively large, in a density curve on the board cross-section that is difficult to interpret. This is because the polymer chips, much larger than the wood chips, are non-uniformly distributed over the cross-section of the manufactured board. The smaller and more homogeneous the polymer chips were, the better the density profile obtained (Figure 2).

**Figure 3:**
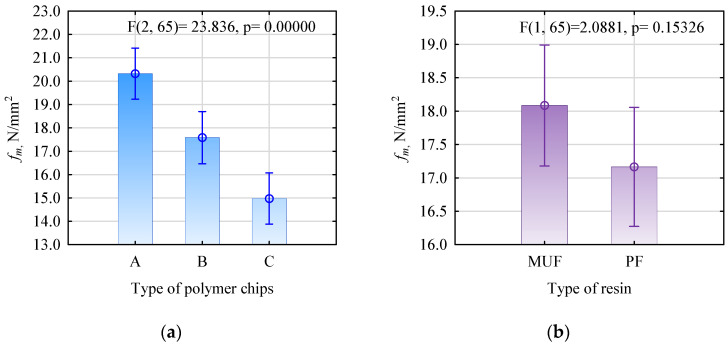
(**a**) Effect of polymer chip type on bending strength of WP boards. (**b**) Effect of resin type on bending strength of WP boards.

The influence of the resin type and polymer chips was analysed based on the static bending strength results. This property is one of the critical characteristics of boards for structural applications. As can be seen from the data shown in Figure 3a, the type of polymer chip influences the static bending strength. The higher the degree of grinding, which was more similar to pine chips and relative to each other, the higher the strength of the boards manufactured. However, there were no statistically significant differences in static bending strength depending on the type of resin used (Figure 3b). The two resins, despite differing chemical structures (aminoplast and phenoplasts), show a very high affinity for bonding to wood.

**Figure 4:**
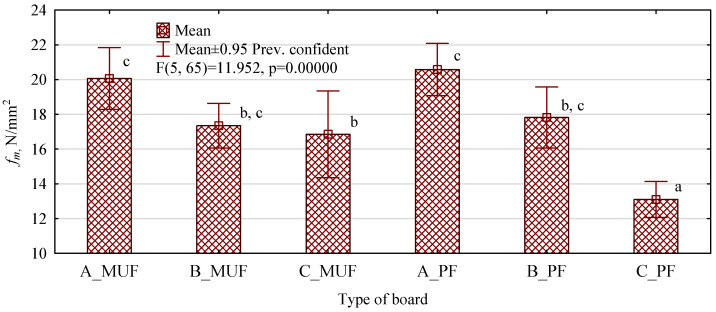
The bending strength of polymer chipboard depending on the type of polymer chip and resin used. Lower-case letters indicate homogeneous groups.

The average static bending strength values of the manufactured particle-polymer boards are shown in Figure 4. From the information presented therein, it can be seen that the boards manufactured using PF resin have significantly lower values than those bonded with MUF resin when C-marked polymer chips were introduced into the particle–glue mixture.

Chips of this type are easy to obtain when recycling rotor blades. However, due to their widely varying linear dimensions and narrow and long particles, they are challenging to distribute uniformly in the bulk of the material input. Furthermore, under laboratory conditions using slow-speed gluers, the uniformity of glue coverage of the chips is significantly lower than in industrial systems based on turbo-gluers. Our practice shows that, with standard chips, the average values obtained in the laboratory usually correspond to the fifth percentile of particleboard produced under the same conditions but on the production line. In this case, this relationship is more difficult to estimate, as the behaviour of chips with a much higher density can vary. They may not interfere with the glue machines and thus be as well covered with glue as wood chips, or they may migrate closer to the surface of the glue machine, reducing the chips’ access to the glue application zone. The quality of the adhesive covering of the chips, among other things, is indicated by the results obtained in the tensile test perpendicular to the board planes.

**Figure 5:**
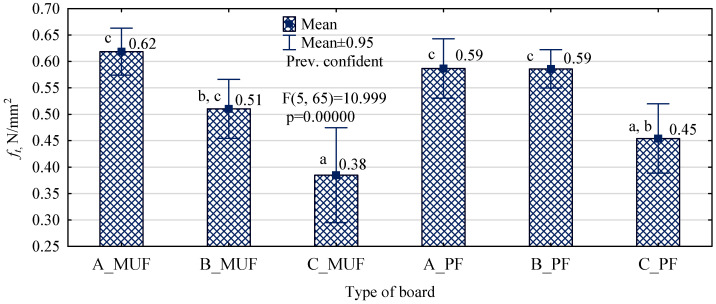
Tensile strength perpendicular to the surfaces of the particleboard depending on the type of polymer chips and resin used (letters mark homogeneous groups).

**Figure 6:**
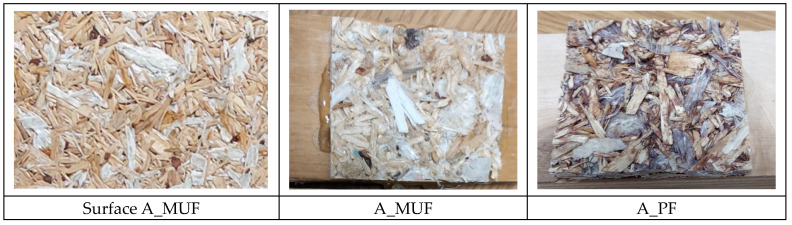
Appearance of the boards after the tensile strength test perpendicular to the surfaces.

However, from the values shown in Figure 5, it can be seen that particleboards manufactured with type A and B polymer chips, regardless of the type of resin used, show similar values. The appearance of the boards after testing is shown in Figure 6. The values determined in the study are high and range from 0.5 N/mm^2^ to 0.6 N/mm^2^. This indicates good adhesive bonding and, due to the not-too-large confidence intervals specified for these boards, good, uniform adhesive coverage of the chips. The situation is different for boards made with type C chips. Although there was no statistically significant difference, lower values were found for type C_MUF boards than for C_PF boards.

Interestingly, the boards made with PF resin showed lower static bending strength values. The highly non-uniform distribution of the polymer chips across the board cross-section may be responsible for this. It is evident from the density profiles determined for the C_MUF boards that some samples were selected for testing in which one side showed a significantly higher density. This distribution of densities may give a bonus to the increased strength of the boards, as defined by the static bending strength and the weaker tensile strength perpendicular to the planes.

Boards made with MUF resin proved to be noticeably stiffer. In their case, high average values were obtained, well above 3500 N/mm^2^. This is the modulus of elasticity declared by the manufacturer of MFP boards, indicating the superiority of its boards over OSB. In the case of manufactured boards, we see an apparent increase in density compared to OSB and MFP. The former has a density of around 650 kg/m^3^, and the latter 750 kg/m^3^. On the other hand, the WP boards in question have a density of around 850 kg/m^3^.

**Figure 7:**
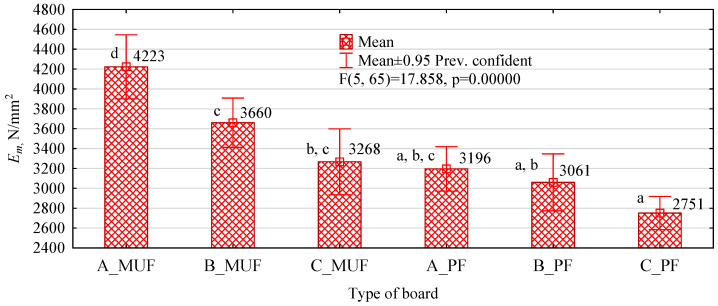
The modulus of elasticity of polymer chipboard depending on the type of polymer chip and resin used (letters mark homogeneous groups).

Nevertheless, it should be remembered that 40% of the input in the analysed boards belongs to polymer chips, which have a density of around 1700–1900 kg/m^3^, i.e., more than three times the assumed density of pine wood (Figure 7).

In the case of type C_MUF boards, although their modulus of elasticity is lower than that of the other types of MUF resin-bonded boards, it is nevertheless slightly, but not statistically significantly, higher than that of PF resin-bonded boards. Only in this area a clear advantage can be seen in the quality of the MUF adhesive bond over PF. This resin, which is the primary bonding agent used in manufacturing these boards, may also be responsible for the high values of the elastic modulus of MFP boards.

**Figure 8:**
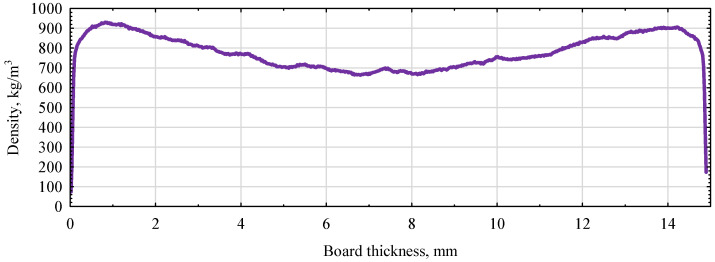
Density profile of MFP boards.

The average modulus of elasticity determined for the industrial boards was 4150 N/mm^2^ (SD 290 N/mm^2^, υ = 7%). The boards had an average density of 780 kg/m^3^ at a moisture content of 6.2%. The high values of the elastic modulus of the MFP boards are probably due to the shape of the density profile, in addition to perhaps the influence of the MUF resin. As can be seen from the data in Figure 8, this type of board has a density profile shape similar to the classic M-profile. The density profile is slightly flatter than three-layer boards, but the surface layers are denser than 900 kg/m^3^.

**Figure 9:**
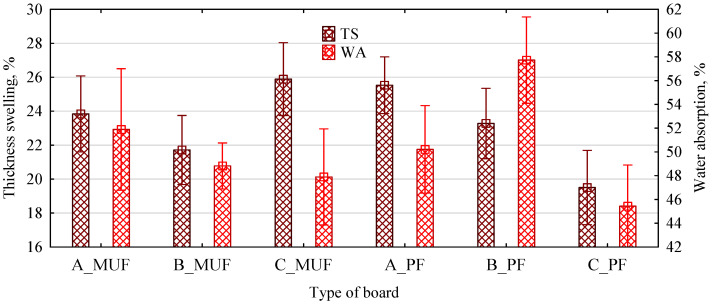
Absorption and swelling of polymer chipboard depending on the type of polymer chip and resin used.

The type of resin used does not have a statistically significant effect on the swelling of the boards so produced (F(1, 65) = 1.8564, *p* = 0.17775). The type of polymer chip also has little effect on the swelling values observed (F(2, 65) = 3.3382, *p* = 0.04168). Although the individual variants differ in their mean values, except for the C_PF boards, the swelling values were between 22% and 26% (Figure 9). Such values should be considered low, without additional water repellents. Nevertheless, the decrease, especially in water absorption, to approx. (42–62)%, must be considered significant. This is because particleboards made from pine chips without additional water repellents usually demonstrate absorption well above 100%.

The low absorbability results in the observed low swelling values. However, due to the assumed wood chip–polymer chip ratios and the observed decreases in hydrophilic characteristics, these are primarily the result of introducing non-water-absorbing material into the board structure. It is essential to recognise that the obtained values for the physical and mechanical characteristics of the manufactured boards indicate the high potential of this method of recycling wind turbine propellers. Sheathing boards often have a much longer life cycle than furniture boards. It can even exceed 50 years. The high stiffness of the manufactured boards fits in very well with current trends in material searching or technological changes, to obtain materials with a high modulus of elasticity, even with increasing their densities. From the data presented in Table 1, it can be seen that panels with densities above 950 kg/m^3^ produced from polymer chips alone, formed from shredded blades bonded with pMDI, show the best mechanical properties. Reducing the degree of bonding results in a noticeable decrease in the mechanical characteristics analysed. The use of thermoplastic polymers, although allowing a relatively high bending strength of the boards to be obtained, results in significantly lower values of the modulus of elasticity. As a result, the boards produced show mechanical properties similar to those made with low and medium degrees of pMDI bonding. The boards we produced achieved good strength results (especially A_MUF), as it is generally accepted that the degree of bonding for pMDI is two to three times lower than for formaldehyde resins. With this relationship, comparable process costs and comparable physical mechanical properties of the particleboards produced are obtained.

## 4. Conclusions

Currently developed technologies aim to reduce the use of valuable or less available raw materials. In the case of wood, the useful, large-sized raw material is directed towards sawmilling. In contrast, medium- and small-sized raw materials and sawmill residues (shavings, sawdust, chips or pieces) are directed towards the panel industry. Nevertheless, efforts are still underway to improve raw material use in particleboard production further by using less common species and other substitutes. Combining wood chips with polymer chips generated from shredding propellers from windmill turbines, boats or other similar items is a good solution.

The conclusions that can be drawn from the study are as follows:

- A mix of 40% polymer and 60% pine chips produces a board with satisfactory characteristics.
- The method of obtaining the polymer chips and their dimensional structure has a much more significant effect on the values obtained (especially on all the mechanical properties) than the adhesive resin used.
- Slightly better properties, essentially linear elastic modulus, were obtained for mela-mine-urea-formaldehyde resin than for phenol-formaldehyde resin.
- In order to maintain the contact between the bonded chips, it is necessary to increase the density of the manufactured boards due to the fact that the material with a much higher density than pine chips is introduced into the chip–glue mixture (which results in a higher bulk density).
- PW boards with fine polymer chips, similar in size to pine chips, achieved significantly higher strengths than boards manufactured with polymer chips, with very different shapes and linear dimensions.

It seems that ground wind turbine blades can be successfully used in industry as a partial substitute for wood chips. The high adhesion of these materials to the adhesives used in the wood-based board industry, which has been confirmed not only in our study, does not pose a technological barrier. There are yet two technological challenges that must be addressed. The first one concerns the degree of shredding and is quite easy to eliminate. The solution is to limit the formation of both very fine silty fractions and fractions much larger than wood chips. The second one is related to the forming of boards, which is done manually under laboratory conditions. Thanks to this procedure, it is easier to distribute the polymer chips evenly between the wood chips. What still remains difficult is assessing how the forming machines, especially the pneumatic ones, will behave.

## Figures and Tables

**Table 1:** Properties of boards containing polymer chips from the shredding of propeller blades in their structure.

Publication	Type of Resin	Variant	Density	*f_m_*	*E_m_*	*f_t_*
			kg/m^3^	N/mm^2^	N/mm^2^	N/mm^2^
Mirski * [46]	UF	2S65	650	12.3	2625	0.57
Mamanpush * [50]	pMDI **	10/5/12.7	1030	40.7	5110	2.34
		6/3/12.7	985	28.9	4215	1.56
		3/5/12.7	1020	21.2	3480	0.82
Mamanpush * [51]	HDPE ***	7	1120	25.0	2030	-
		23	1150	24.2	2072	-
		25	1095	23.3	1928	-

* Co-authored publications, ** pMDI/MDI 4,4-diphenylmethane diisocyanate, *** HDPE—high density polyethylene.

## Data Availability

Data available on request due to large size and complicate structure.
